# Optical Aberration Calibration and Correction of Photographic System Based on Wavefront Coding

**DOI:** 10.3390/s21124011

**Published:** 2021-06-10

**Authors:** Chuanwei Yao, Yibing Shen

**Affiliations:** State Key Laboratory of Modern Optical Instrumentation, Zhejiang University, Hangzhou 310027, China; 21930003@zju.edu.cn

**Keywords:** image processing, PSF, wavefront coding, deconvolution, photographic system

## Abstract

The image deconvolution technique can recover potential sharp images from blurred images affected by aberrations. Obtaining the point spread function (PSF) of the imaging system accurately is a prerequisite for robust deconvolution. In this paper, a computational imaging method based on wavefront coding is proposed to reconstruct the wavefront aberration of a photographic system. Firstly, a group of images affected by local aberration is obtained by applying wavefront coding on the optical system’s spectral plane. Then, the PSF is recovered accurately by pupil function synthesis, and finally, the aberration-affected images are recovered by image deconvolution. After aberration correction, the image’s coefficient of variation and mean relative deviation are improved by 60% and 30%, respectively, and the image can reach the limit of resolution of the sensor, as proved by the resolution test board. Meanwhile, the method’s robust anti-noise capability is confirmed through simulation experiments. Through the conversion of the complexity of optical design to a post-processing algorithm, this method offers an economical and efficient strategy for obtaining high-resolution and high-quality images using a simple large-field lens.

## 1. Introduction

The performance of optical systems depends considerably on the design of the optical system, as aberration is a key obstacle for an optical system to reach the ideal diffraction-limited resolution. To obtain high-quality images, optical imaging systems designers must correct and balance aberrations by combining multiple lenses of different glass materials. Even if the final design of the optical systems meets the requirements, it will make optical systems cumbersome and expensive.

Fortunately, the aberration correction problem can be reshaped into a computational problem to be solved after the acquisition of the image data. The acquired aberrated image is digitally post-processed using an image deconvolution algorithm to reconstruct an aberration-corrected high-quality image [1]. This lowers the expense and complexity of the optics while ensuring the resolution of the optical system [2]. The blur kernel used in the deconvolution calculation—i.e., the optical system’s point spread function (PSF)—is a key factor in determining the image reconstruction’s quality. If the PSF is not obtained accurately, the reconstructed images are most likely to have severe artifacts and ringing effects [3,4], affecting the quality of the image reconstruction.

In recent years, a few methods have been proposed to correct optical aberrations and improve image quality by acquiring PSFs. The blind deconvolution algorithm is a widely used method for PSF acquisition [5,6,7,8] that uses prior knowledge to estimate a clear image and the PSF directly from the blurred image by minimizing the cost function. However, the PSF of optical systems is always spatially variant and needs to be processed in patches. However, the image information in each small patch is very limited, and the lack of information will cause the estimation of PSF to be inaccurate. The restoration results are not reliable. The most intuitive PSF acquisition method is the direct measurement method [9] which can directly obtain the optical system’s impulse response to the point light source; i.e., PSF. However, the light intensity of the point light source is weak, the measurement results are subject to sensor noise, and the signal-to-noise ratio is low. The fitted parameter method [10,11] uses the measured PSF to match the simulated PSF to calibrate the lens prescription and then compute fitted PSFs by simulation. However, for optical systems with low mounting accuracy, system mounting errors can have a serious impact on the PSF estimation.

Another way to mitigate optical aberrations operates by adding masks to the optical system to encode the wavefront, including amplitude masks [12,13] and phase masks [14,15,16]. However, amplitude masks can block a portion of the incident light, and very fine printing patterns can cause diffraction artifacts [12]. Meanwhile, phase masks reduce the effective imaging resolution of the imaging system [15].

Another class of methods acquires the optical system’s PSF by reconstructing the wavefront. A simple method is to employ the Shack–Hartmann wavefront sensor [17], which consists of a lenslet array and an array detector. The phase of the wavefront can be linked to the local focal spot shifts in the corresponding region. Despite its simple principle, significant setup modifications are unavoidable. The density of the microlens limits its spatial resolution and sensitivity, so the phase approximation is rough. Another class of methods reconstructs the wavefront directly from intensity measurements by using a phase retrieval procedure. This method first introduces phase diversity in the multiple optical field intensity patterns recorded by the camera and then uses an iterative algorithm to reconstruct the wavefront from the recorded intensity patterns. One simple way to introduce phase diversity is by defocusing (i.e., axial scanning). There are a variety of reported methods for phase retrieval using defocus diversity, including iterative algorithms [18], transport-of-intensity equation (TIE)-based methods [19,20], and other non-iterative methods [21]. However, since the optical resolution can exceed the pixel resolution of the detector easily, these methods are susceptible to pixelation artifacts. Other approaches use wavefront coding to introduce phase diversity [22,23,24]. Those methods reconstruct the wavefront of the Fourier ptychography microscope (FPM) system by utilizing the redundancies in the dataset acquired by FPM to eliminate the effects of aberrations in the FPM system on the reconstruction results. However, since the spatial light modulator’s (SLM) angle-dependent amplitude response has less variation only at low-incidence angles [25], the wavefront’s reconstruction results with large field of view (FOV) setups can be bounded by this effect. To date, few studies have reported on the aberration correction of a photographic system based on wavefront coding.

**Figure 1:**
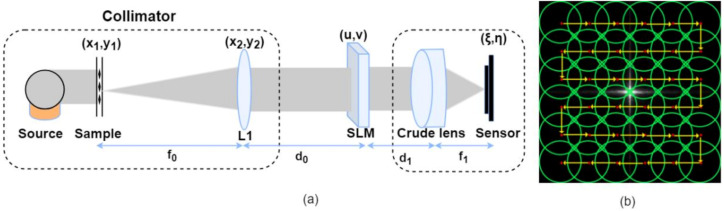
(**a**) The experimental setup schematic. (**b**) The sub-aperture M(u,v) loaded on the SLM. Green circles indicate the coverage of each sub-aperture. Red dots indicate the center position of the sub-aperture. Yellow arrows indicate each movement of the sub-aperture.

Here, we perform an optical aberration calibration and correction method of photographic systems based on wavefront encoding. To minimize the aberrations introduced by optical elements besides the given lens during the measurement process, we designed an experimental setup resembling the direct measurement method, as shown in Figure 1. The experimental setup allowed us to accurately calibrate the PSF of a given photographic lens at an arbitrary FOV. However, in contrast to the direct measurement method using the point source images taken with the given lens to obtain the PSF, our method acquires the PSF from multiple images obtained by wavefront encoding. Thus, our method is less demanding in terms of the light source brightness, target shape, and sensor, and more resistant to sensor noise, resulting in more robust PSF calibration results.

## 2. Materials and Methods

Similar to the direct measurement method’s experimental setup, as shown in Figure 1a, the prototype system design consists of a collimator, an SLM, the crude optical system that requires aberration correction, and the sensor element. The object is first imaged to infinity by the collimator. Due to the collimator’s long focal length, the light has a small incident beam angle and can be approximated as a near-axis light without aberration introduced. The light wavefront is then modulated by the SLM and is recorded by an image sensor after passing through the crude optical system.

For an optical system with a large FOV, the aberration is spatially varied. We divide the full FOV into multiple small fields of view. Within a smaller field of view angle, it can be assumed that the spatially varying aberrations are invariant [26,27,28]. In the following discussion, we constrain our analysis to a particular FOV.

We consider an unknown sample s(x,y) located in the field-of-view range $t_{0}$. A point source $\left(x_{0},y_{0}\right)$ with an amplitude and phase *C* on the sample plane can be described by

(1) $$E_{1}(x_{1},y_{1})=C\delta (x_{1}-x_{0},y_{1}-y_{0})$$

The wavefront transmits by Fresnel propagation to the front surface of $L_{1}$:

(2) $$\begin{array}{cc} E_{2}(x_{2},y_{2})= & \frac{C\exp(\frac{j\pi }{\lambda f_{0}}(x_{2}^{2}+y_{2}^{2}))}{j\lambda f_{0}}\int_{-\infty }^{+\infty }\delta (x_{1}-x_{0},y_{1}-y_{0})\exp(\frac{-j\cdot 2\pi }{\lambda f_{0}}(x_{1}x_{2}+y_{1}y_{2}))\exp(\frac{j\pi }{\lambda f_{0}}(x_{1}^{2}+y_{1}^{2}))dx_{1}dy_{1}\\ & =\frac{C\exp(\frac{j\pi }{\lambda f_{0}}(x_{2}^{2}+y_{2}^{2}))}{j\lambda f_{0}}\exp(\frac{-j\cdot 2\pi }{\lambda f_{0}}(x_{0}x_{2}+y_{0}y_{2}))\exp(\frac{j\pi }{\lambda f_{0}}(x_{0}^{2}+y_{0}^{2})) \end{array}$$

The idealized thin lens that has a focal length $f_{0}$ causes a phase delay of $\exp(-j\frac{\pi }{\lambda f_{0}}(x_{2}^{2}+y_{2}^{2}))$, so the distribution of the light field after passing through the $L_{1}$ is

(3) $$\begin{array}{ll} E_{2}^{\prime }(x_{2},y_{2}) & =E_{2}(x_{2},y_{2})⊗\exp(\frac{-j\pi }{\lambda f_{0}}(x_{2}^{2}+y_{2}^{2}))\\ & =\frac{C}{j\lambda f_{0}}\exp(\frac{-j\cdot 2\pi }{\lambda f_{0}}(x_{0}x_{2}+y_{0}y_{2}))\exp(\frac{j\pi }{\lambda f_{0}}(x_{0}^{2}+y_{0}^{2})) \end{array}$$

The field then propagates with distance $d_{0}$ to reach the spectral plane, introducing a frequency-dependent phase factor $e^{-jkd_{0}\sqrt{1-(\cos^{2}\alpha +\cos^{2}\beta )}}$, and the field can be expressed as

(4) $$E_{3}(u,v)=F^{-1}\{F\{E_{2}^{\prime }(x_{2},y_{2})\exp(-jkd_{0}\sqrt{1-(\cos^{2}\alpha +\cos^{2}\beta )})\}\}$$

Subsequently, a mask M(u,v) is applied to the field, while any discrepancies between the imaging system and the ideal are included in the pupil function P(u,v). The field expression can be obtained as

(5) $$E_{3}^{\prime }(u,v){=E}_{3}(u,v)P(u,v)M(u,v)$$

The field distribution at the sensor plane is

(6) $$\begin{array}{ll} E_{4}(\xi ,\eta ) & =\frac{\exp([\frac{ik}{2f_{1}}(1-\frac{d_{1}}{f_{1}})(\xi^{2}+\eta^{2})])}{j\lambda f_{1}}\cdot {F\{E}_{3}^{\prime }(u,v)\}(\xi ,\eta )\\ & =A\cdot F\{P(u,v)M(u,v)\}(\xi ,\eta )⊗\delta (\xi +\frac{x_{0}}{\lambda f_{0}},\eta +\frac{y_{0}}{\lambda f_{0}})\\ & =A\cdot F\{P(u,v)M(u,v)\}(\xi +\frac{x_{0}}{\lambda f_{0}},\eta +\frac{y_{0}}{\lambda f_{0}}) \end{array}$$

Setting $A=\frac{C\exp(j\frac{\pi }{\lambda f_{0}}(x_{0}^{2}+y_{0}^{2}))}{j\lambda f_{0}}\frac{\exp([\frac{ik}{2f_{1}}(1-\frac{d_{1}}{f_{1}})(\xi^{2}+\eta^{2})])}{j\lambda f_{1}}\exp(-jkd_{0}\sqrt{1-\lambda^{2}(\xi^{2}+\eta^{2})})$ where $E_{4}(\xi ,\eta )$ is the complex field that is incident on the sensor after the point source located at $\left(x_{0},y_{0}\right)$ has passed through the optical system. It is the point spread function of the optical system. Since our imaging system is incoherent, the phase relations between points in the sample plane are not relevant, and the complex phase fluctuations in A related to $d_{0},d_{1}$ are irrelevant and have no effect on the captured images. The imaging system’s intensity PSF can be defined as

(7) $$h(\xi +\frac{x_{0}}{\lambda f_{0}},\eta +\frac{y_{0}}{\lambda f_{0}})={\left|E_{4}(\xi ,\eta )\right|}^{2}={\left|A\right|}^{2}\cdot {\left|F\{P(u,v)M(u,v)\}(\xi +\frac{x_{0}}{\lambda f_{0}},\eta +\frac{y_{0}}{\lambda f_{0}})\right|}^{2}$$

Thus, after neglecting the constants and dropping coordinate scaling,

(8) $$h(\xi ,\eta )={\left|F\{P(u,v)M(u,v)\}(\xi ,\eta )\right|}^{2}$$

In a small field of view range $t_{0}$, the aberration can be considered spatially invariant. For a particular aperture mask M(u,v), h(ξ,η) is the intensity of the optical system’s PSF. The image $i_{t0}(\xi ,\eta )$ of an unknown sample S(x,y) captured by the sensor plane for an aperture mask M(u,v) can be expressed as

(9) $$i_{t0}(\xi ,\eta )=h_{to}(\xi ,\eta )⊗|S(\xi ,\eta ){|}^{2}=h_{t0}(\xi ,\eta )⊗l(\xi ,\eta )$$

where l(ξ,η) is the intensity of S(ξ,η). The equation shows that the sample’s captured image is subject to the effect of the PSF derived from the sub-regions of the pupil.

By scanning the Fourier spectrum of a sample, we can use the captured intensity images to synthesize the sample’s Fourier spectrum by the phase retrieval algorithm. In our work, the PSFs are the intensity images captured by the sensor, and the pupil function is the Fourier spectrum to be synthesized. Thus, we can use the phase retrieval algorithm to synthesize the pupil function using a series of acquired PSF intensities by scanning the optical system’s spectral plane. The aberrations in the image are then removed by deconvolution.

**Figure 2:**
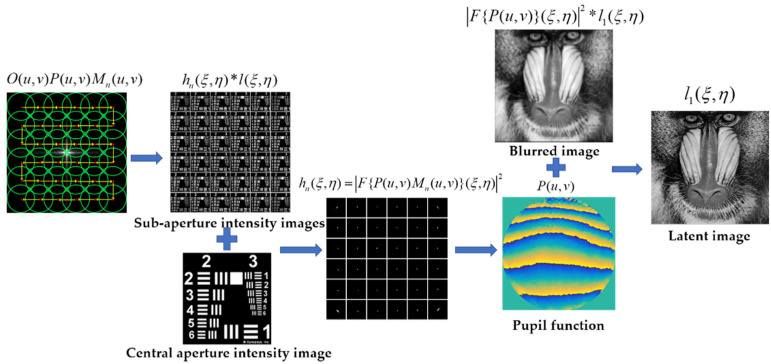
The framework of the proposed method.

As shown in Figure 2, we calibrate and correct the optical system for aberrations in three steps: (1) firstly, we obtain *n* sets of intensity images $i_{n}(s,t)$ by moving the sub-aperture $M_{n}(u,v)$ sequentially through the *n* sub-aperture positions in the spectral plane, as shown in Figure 1b. Then, we perform local aberration recovery to obtain the *n* PSF intensities $h_{n}(s,t)$ determined by the *n* masks in the spectral plane; (2) we reconstruct the pupil function P(u,v) from the obtained *n* PSF intensities $h_{n}(s,t)$ using a phase recovery algorithm; and (3) the reconstructed pupil function P(u,v) is used to deconvolve the aberrated image i(s,t) acquired by the crude optical system to obtain the aberration-free image l(s,t). 

The following three subsections present these steps in detail.

### 2.1. Local Aberration Recovery

To obtain the point spread function $h_{n}(s,t)$ desired to reconstruct the pupil function P(u,v), we first obtained *n* sets of intensity images $i_{n}(s,t)$ by applying a sub-aperture $M_{n}(s,t)$ at *n* locations in the spectral plane. We then applied an image pair-based blur kernel estimation algorithm [29] to determine the local aberration, in which one of the image pairs was assumably blur-free, whereas the other image was aberration-blurred. In general, the center of an imaging lens can be regarded as a region that is free from aberrations that can reach a diffraction-limited resolution. The image $i_{1}(s,t)$ obtained with the central aperture can be used as a reference image to determine the pupil function of the entire aperture, and the differences between this reference image and the images $i_{n}(s,t)$ of the sub-aperture at other locations are attributed to the residual aberration [26]. We estimate the local aberration PSF using an iterative Tikhonov deconvolution in the Fourier domain [30].

The update of $h_{n}\left(s,t\right)$ in the Fourier domain is given by

(10) $$H_{n}^{k+1}(u,v)=H_{n}^{k}(u,v)+\beta \frac{\left|I_{1}(u,v)|I_{1}^{*}(u,v)(I_{n}^{\prime }(u,v)-I_{1}(u,v)H_{n}^{k}(u,v)\right)}{\left|I_{1}(u,v){|}_{\max}(|I_{1}(u,v){|}^{2}+\alpha \right)}$$

where $H_{n}(u,v)$ and $I_{n}(u,v)$ are the Fourier spectra of $h_{n}(s,t)$ and $i_{n}(s,t)$, respectively, β is the scaling constant to adjust the iteration step, and α is a small value to ensure the stability of the value during the iteration.

**Figure 3:**
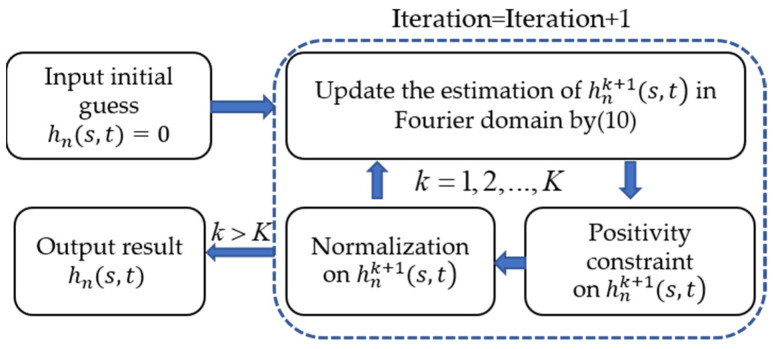
The algorithm flow chart of the local aberration recovery process.

The algorithm flow is depicted in Figure 3.

With this algorithm, we can obtain the local PSF and the intensity information of the pupil function captured by the sensor plane under the modulation of *n* sub-apertures. We show the synthesis of the complete pupil function by the phase retrieval algorithm in the next section.

### 2.2. Pupil Function Reconstruction

**Figure 4:**
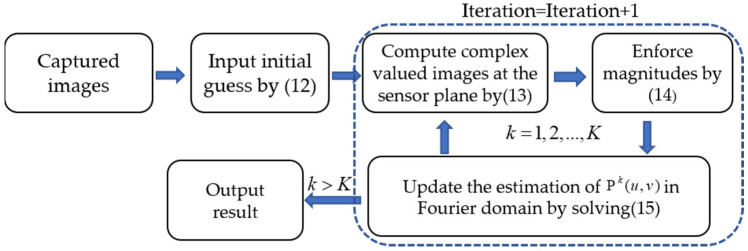
The algorithm flow chart of the pupil function reconstruction process.

By scanning the Fourier spectrum of the sample, the phase retrieval algorithm can synthesize the sample’s Fourier spectrum from a collection of intensity images captured by the sensor. In this paper, the pupil function is the Fourier spectrum we want to recover, and the *n* PSFs obtained by the local aberration recovery algorithm in the previous part are the intensity images used for reconstruction. To reconstruct the optical pupil function, we used an alternating minimization-based phase retrieval algorithm [31], and the algorithm flow is given in Figure 4.

We aimed to solve the following problem:

(11) $$P^{*}(u,v)=\underset{P(u,v)}{\arg\min}\sum_{n}∥{\hat{p}}_{n}(s,t)-{F\{M}_{n}\left(u,v\right)\cdot P(u,v)\}{∥}_{2}s.t.{\left|{\hat{p}}_{n}(s,t)\right|}^{2}=h_{n}(s,t)$$

At each iteration (k), we performed the following three steps to update the estimate of the pupil function:

(1): $h_{1}(s,t)$ is the PSF intensity captured by the sensor with the central aperture. We set the scaled version of $F\{\sqrt{h_{1}(s,t)}\}(u,v)$ to the initial estimate of P(u,v) and subsequently used the estimate of the optical pupil function to calculate the complex-valued field ${\hat{p}}_{n}^{k}(s,t)$ at the sensor:

(12) $$P^{1}(u,v)=F\{\sqrt{h_{a,1}(s,t)}\}(u,v)$$

(13) $${\hat{p}}_{n}^{k}(s,t)=F\{M_{n}\left(u,v\right)\cdot P^{k}(u,v)\}$$

(2) We replaced the amplitude of ${\hat{p}}_{a,n}^{k}(u,v)$ with the amplitude of the corresponding actual PSF $h_{n}(s,t)$:

(14) $${\hat{p}}_{a,n}^{k}(s,t)\leftarrow \sqrt{\frac{h_{n}(s,t)}{{\left|{\hat{p}}_{a,n}^{k}(s,t)\right|}^{2}}}\cdot {\hat{p}}_{a,n}^{k}(s,t)$$

(3) We updated the estimate of $P^{k}(u,v)$ by solving the following regularized, least-squares problem:

(15) $$P^{k+1}(u,v)\leftarrow \underset{P(u,v)}{\min\mathrm{imize}}\sum_{n}{∥{\hat{p}}_{n}^{k}(s,t)-F\{M_{n}\left(u,v\right)\cdot P(u,v)\}∥}_{2}^{2}+\tau {∥P(u,v)∥}_{2}^{2}$$

where τ>0 is the regularization parameter used to ensure numerical stability during the reconstruction [32]. This regularized, least-squares problem has a fixed form solution that can be efficiently computed by the fast Fourier transform.

### 2.3. Image Deconvolution

By using the algorithm in the previous section, we were able to obtain the pupil function P(u,v) of the crude optical system, which in turn gave us the PSF of the optical system; i.e., $h(s,t)={\left|F\{P(u,v)\}(s,t)\right|}^{2}$. We could therefore recover the latent image l(s,t) from the aberrated image i(s,t).

In an optical system, the latent image l(s,t) is blurred by the PSF h(s,t). The intensity image obtained at the sensor can be denoted as i(s,t)=h(s,t)⊗l(s,t)+n(s,t), where n(s,t) is the additive noise and ⊗ is the convolution operator. To suppress the effect of noise on the deconvolution result, we used a deconvolution method employing regularization [33] to obtain the latent image l(s,t).

## 3. Experiments and Results

To certify the effectiveness of the proposed method, we conducted both simulated and real data experiments.

### 3.1. Simulated Data Experiments

**Figure 5:**
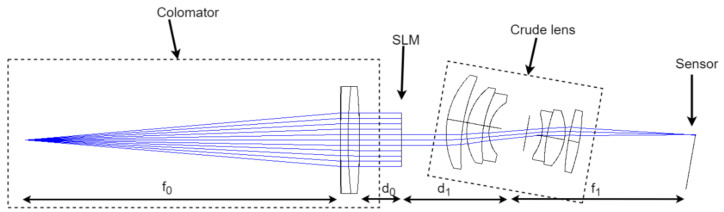
Simulated experimental setup built in CODEV.

In this paper, we used the imaging simulation function of the CODEV software to investigate the effectiveness of the proposed method for reconstructing the PSF. Firstly, we built the optical system shown in Figure 5 in CODEV, including a long-focus double-glued lens as a collimator (D_0_ = 50.8 mm, f_0_ = 500 mm, GCL-010611), an SLM, and the crude optical system. The focal length of the crude optical system was $f_{1}=75\;\mathrm{mm}$, and the pupil diameter was $D_{1}=25\;\mathrm{mm}$.

**Figure 6:**
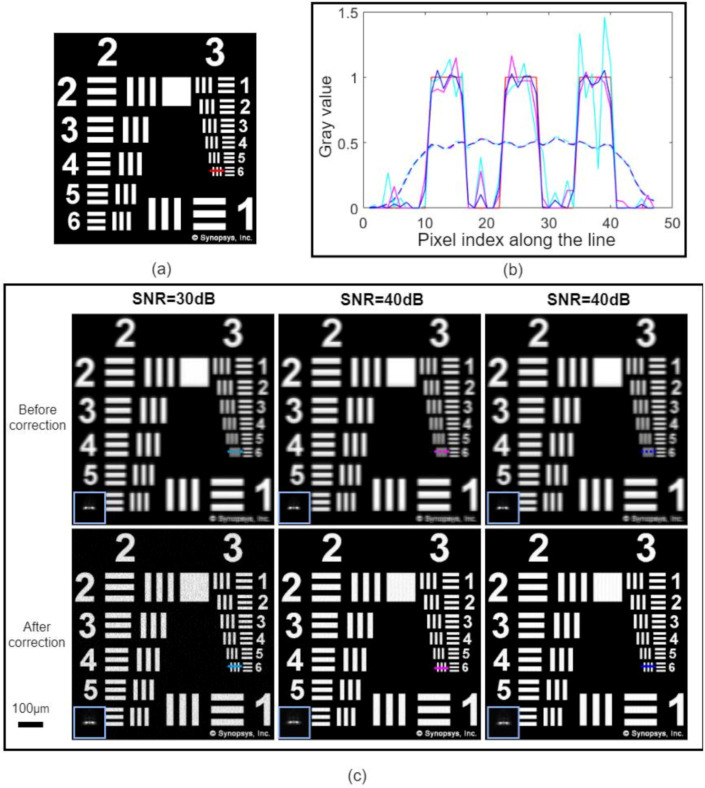
Simulation results of aberration correction: (**a**) original image reprinted with permission from Synopsis, Inc., (**b**) the line outline of the periodic line in (**c**). (**c**) The results before and after the correction of images with different noise levels are compared, and the inset boxes show the used measured PSF.

Since the given optical system was not well-corrected, it had severe off-axis aberrations. The crude optical system was rotated 10° to reconstruct the PSF at the 10°field of view of the crude optical system. A series of sub-apertures were loaded on the SLM plane as shown in Figure 1b, the diameter of the sub-apertures was d=5 mm, and the distance of each shift was δ=3.8 mm. At each sub-aperture position, the sub-aperture images were obtained using the software’s image simulation function, and then 30 dB,40 dB,50 dB of Gaussian noise was added, respectively. The reconstructed PSFs obtained using the proposed method are shown in the blue inset boxes in Figure 6(c4–c6).

We used the 664 px × 664 px resolution chart in Figure 6a as the original image. Then, we generated the blurred image at the 10° FOV of the crude optical system using the imaging simulation function of the CODEV and added 30 dB,40 dB,50 dB, if Gaussian noise to the image, respectively, as shown in Figure 6(c1–c3). The original PSF of the crude optical system is shown in the blue inset box. Finally, we conducted aberration correction using the reconstructed PSF. The image comparison before and after aberration correction is shown in Figure 6c. It can be seen that our method was very effective in reconstructing the PSF and in removing aberrations from the blurred images at variable noise levels. The aberration-corrected image had sharper edges, significantly better definition, and no ringing effect. As indicated by the line profile in Figure 6b, the aberration-corrected lines were still well resolved even in the presence of severe noise.

The availability of truth data in simulated experiments allowed us to further quantitatively evaluate image quality. The full reference indexes—the structural similarity index (SSIM), and peak signal-to-noise ratio (PSNR) [34,35]—were utilized to assess the similarity between the image before and after aberration correction and the original image. Higher values of PSNR and SSIM indicated that the image was more similar to the original image and that the image recovery was more effective.

The quantitative evaluation results of the simulated experiments are shown in Table 1. Although the aberration could be effectively corrected when the image signal-to-noise ratio was 30 dB, the evaluation index was not greatly improved due to severe noise. The evaluation indexes of the remaining corrected images were greatly improved, which was consistent with the visual evaluation effect.

In summary, the proposed method was able to reconstruct the PSF of the optical system robustly. The aberration-corrected images obtained by the proposed method were able to remove the aberration well without the ringing effect. We could also verify that our method has good noise immunity and can accurately reconstruct the PSF of an optical system in the presence of severe noise.

### 3.2. Real Data Experiments

**Figure 7:**
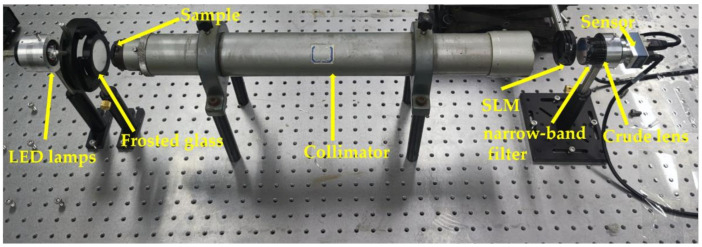
Experimental Setup.

Our experimental setup was similar to the simulated optical system, as shown in Figure 7, where we used a collimator with focal length $f_{0}=500\;\mathrm{mm}$ and aperture D=50.8 mm. The crude optical system consisted of an inexpensive industrial lens $\left(f_{1}=50\;\mathrm{mm},F/3.3\right)$ and a narrow-band filter (λ=0.54 μm. The industrial lens was focused on optical infinity, and this large F-number industrial lens had significant off-axis aberrations due to being improperly corrected. We established a shifting sub-aperture mask of the spectral plane by using a physical iris with an adjustable aperture fitted on a two-dimensional translation stage. 

In our experiments, the range of the scanned spectral plane was matched to the pupil diameter R of the optical system. The position of the sub-aperture was determined by the radius r of the sub-aperture and the overlap δ between adjacent sub-apertures; i.e., $2R=2r+\left(n-1\right)*\left(2r-\delta \right)$, where n is the number of sub-apertures in each column. Firstly, the radius of the sub-aperture should make the image data oversampling rate $S=\frac{\lambda f_{1}}{2r\cdot r_{CCD}}$ greater than 2. Furthermore, a smaller sub-aperture radius results in a more accurate wavefront reconstruction. However, a smaller sub-aperture radius implies a multiplication of the number of sub-apertures. A smaller number of sub-apertures is desired without going under the sampling limit. Thus, a sub-aperture radius of r=1.8 mm was a balanced choice in our experiments. Our method was not affected greatly by the overlap rate between adjacent sub-apertures, and when the overlap rate $\eta =\frac{\delta }{2r}\cdot 100\%$ between sub-apertures was zero, the results of our method became very similar to those of the Shack–Hartmann sensor. Therefore, we chose the minimum overlap rate η=16.25% required to enable complete coverage of the spectral plane by the sub-aperture.

In our experiments, we moved the sub-aperture n=6 times in sequence laterally in x and y directions through a region with a L×L=15.3 mm×15.3 mm square spectral plane. In every step, we moved the sub-aperture by δ=2.34 mm until it crossed all $n^{2}=36$ distinct sub-aperture locations. We took multiple snapshots and combined them via high dynamic range processing (HDR) [36] at each sub-aperture position to ensure that each image photographed was properly exposed.

In the image plane, we placed a CCD sensor (Sony IMX253 Genie Nano CL-M4040 from Teledyne Dalsa in Waterloo, Canada, with an effective resolution of 4112 pixels × 3008 pixels (14 mm × 10 mm) and a pixel size $r_{CCD}=3.45\;\mu m$). The image data oversampling rate was $S=\frac{\lambda f_{1}}{r_{CCD}d}>2\;Sa/s$, which thus satisfied the Nyquist sampling requirement [37].

To quantitatively verify the reliability of the results obtained after aberration correction, we first conducted experiments using a WT1005-62 resolution test target as a sample. In our experiments, we first imaged the resolution test target through a collimator to optical infinity. Subsequently, we rotated the crude optical system to collect data from multiple fields of view.

**Figure 8:**
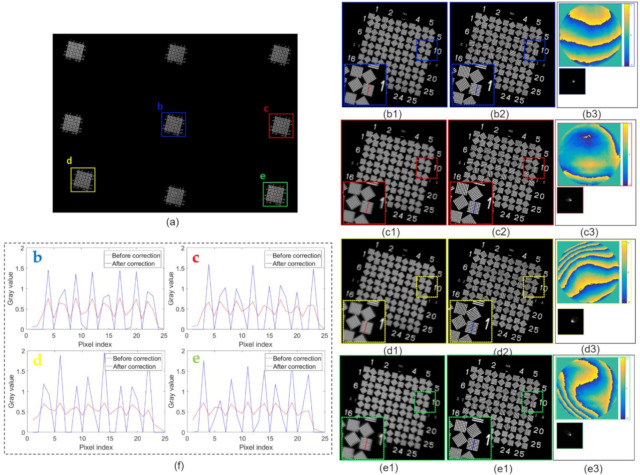
Spatially varying aberration calibration and correction result on a WT1005-62 resolution test target. (**a**) Full FOV image. The pupil function and PSF of each small region denoted by (**b1**–**e1**) varied spatially, as shown in (**b3**–**e3**). The deconvolution results (**b2**–**e2**) show that the spatially varying aberrations were adequately corrected after processing. The inset frame enlarges part of the sample to indicate the enhanced resolution. (**f**) Line profiles through the line pairs in (**b1**–**e1**) (**b2**–**e2**) to highlight the aberration correction performance.

Following the scheme mentioned in Section 2, the image comparison before and after aberration correction is presented in Figure 8. Figure 8a shows the distribution of the measured data sets in the whole field of view. Figure 8(b1~e1) and Figure 8(b2~e2) show the images of four different fields of view before and after the aberration correction, respectively. The local part of the resolution target is enlarged and shown in the inset box to show the resolution improvement. It can be seen that the images before correction had serious off-axis aberrations. The aberrations were well corrected across all fields of view after deconvolution, and no artifacts were produced. Simultaneously, it can be noticed that Group 15 ($d_{\mathrm{width}}=3.56\;\mu m$) was well-resolved after aberration correction, corresponding to the resolution limit of the sensor.

To further quantify the resolution improvement before and after aberration correction, the line profile of line sections before and after aberration correction is shown and compared in Figure 8f. The red and blue curves are the line profile curves before and after correction, respectively. The contrast between the test target lines before correction was very low. After compensation, the peaks of the test target were more evenly spaced, and the improved contrast between the peaks and the troughs verified the usefulness of our aberration correction method.

Two non-reference indexes—the coefficient of variation (CV) and the mean relative deviation (MRD) [38]—were used in the real experiments for quantitative evaluation, and the results are presented in Table 2. Here, it can be seen that CV values and MRD values increased on average by 60% and 30%, respectively, in real experiments, implying the improvement of image sharpness and confirming the feasibility of the proposed method.

**Figure 9:**
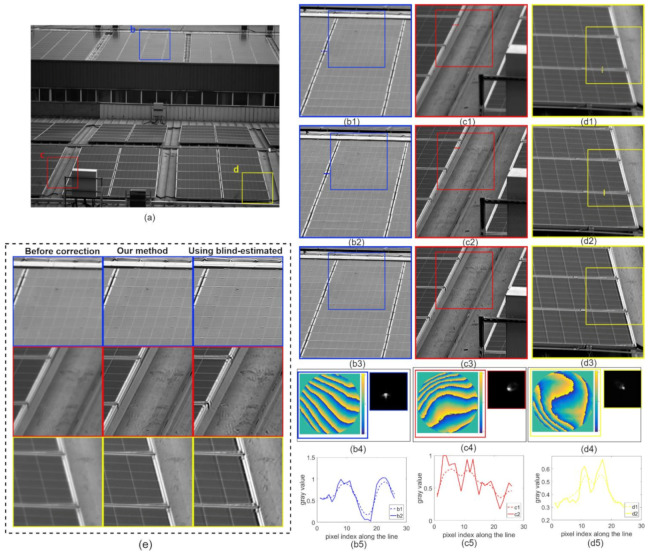
Results for the factory image. (**a**) Full FOV image captured by our inexpensive industrial lens. The pupil function and PSF of each small region denoted by (**b1**–**d1**) varied spatially, as shown in (**b4**–**d4**). The deconvolution results (**b2**–**d2**) show that the spatially varying aberrations were adequately corrected after processing. (**b3**–**d3**) The restored results using blind-estimated PSFs. (**b5**–**d5**) Line profiles at the underlined places in (**b1**–**d1**) (**b2**–**d2**) to show the improved image sharpness before and after correction using the proposed method. (**e**) The three images on the left are enlargements of the inset frames in (**b1**–**d1**), which are captured as blurred images in different FOV. The three images in the middle are enlargements of the inset frames in (**b2**–**d2**) and are the restored results corrected using the proposed method. The three images on the right are enlargements of the inset frames in (**b3**–**d3**), the results of the correction using the blind-estimation method.

**Figure 10:**
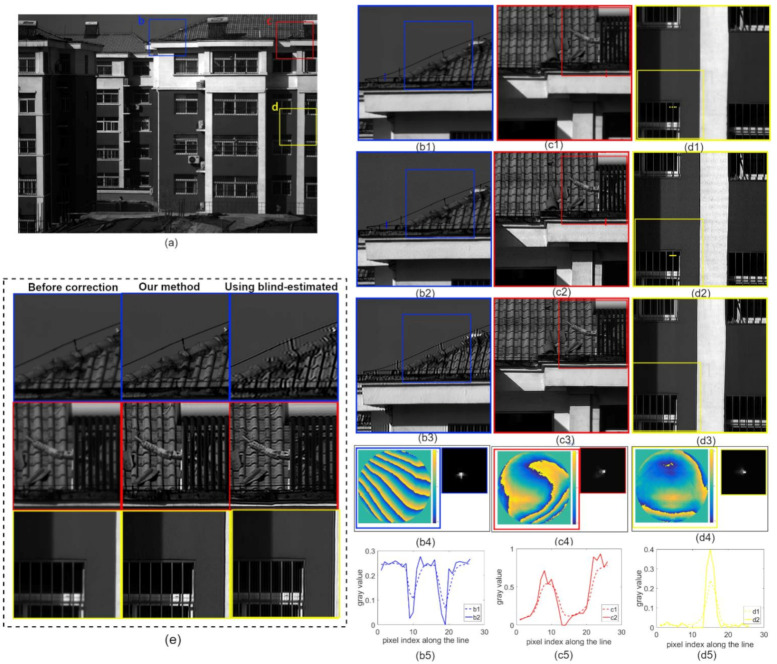
Results for the building image. (**a**) Full FOV image captured by our inexpensive industrial lens. The pupil function and PSF of each small region denoted by (**b1**–**d1**) varied spatially, as shown in (**b4**–**d4**). The deconvolution results (**b2**–**d2**) show that the spatially varying aberrations were adequately corrected after processing. (**b3**–**d3**) The restored results using blind-estimated PSFs. (**b5**–**d5**) Line profiles at the underlined places in (**b1**–**d1**) (**b2**–**d2**) to show the improved image sharpness before and after correction using the proposed method. (**e**) The three images on the left are enlargements of the inset frames in (**b1**–**d1**), which are captured as blurred images in different FOV. The three images in the middle are enlargements of the inset frames in (**b2**–**d2**) and are the restored results corrected using the proposed method. The three images on the right are enlargements of the inset frames in (**b3**–**d3**), the results of the correction using the blind-estimation method..

To further validate our method’s ability to correct aberrations when imaging complex scenes, Figure 9 and Figure 10 show real-world images captured by our inexpensive industrial lens. Figure 9 and Figure 10(b1~d1) show the enlarged images of the contents in the corresponding color boxes in Figure 9 and Figure 10a, respectively. Figure 9 and Figure 10(b2~d2) show the images after aberration correction using our method. After aberration correction, the sharpness of the image was significantly improved, as shown by the line profiles in Figure 9 and Figure 10(b5~e5). Meanwhile, we restored the image patches with the blind-estimated PSF [39] for comparison, as shown in Figure 9 and Figure 10(b3~d3). The deconvolution method and deconvolution parameters were the same as in our proposed method. The deconvolution using the blind-estimated PSF resulted in significant artifacts, and thus could not reasonably handle the images captured using the inexpensive industrial lens. Compared to the blind method, our proposed method not only removed the aberrations robustly but also produced almost no artifacts, and the quality of images was more stable.

## 4. Discussion

In this paper, we propose a method for measuring PSF based on wavefront coding. By performing wavefront coding, local aberration recovery, and pupil function reconstruction, we can reconstruct the crude photographic system’s pupil function accurately. We designed simulated and real experiments for our experiments, and both qualitative and quantitative assessments confirmed that the proposed method can precisely reconstruct the given photographic system’s PSF across all fields of view under different noise conditions, which shows that our method has good universality. Finally, we can obtain high-quality and aberration-free images by performing deconvolution.

Although our method works robustly on reconstructing the PSF, the method still suffers from the limitation of long measurement times. Thus, in the future, we will focus on reducing data acquisition times; for example, by using faster SLM and better scanning strategies.

## Figures and Tables

**Table 1:** Quantitative assessment of simulated data experiments.

Index	Images	SNR = 30 dB	SNR = 40 dB	SNR = 50 dB
PSNR	Before correction	15.5182	15.5333	15.5348
	After correction	17.6292	25.8131	29.6136
SSIM	Before correction	0.5558	0.6056	0.6110
	After correction	0.5621	0.7932	0.9164

**Table 2:** Quantitative assessment of the real data experiments.

Index	Images	b	c	d	e
CV	Before correction	1.5536	1.3910	1.3207	1.3313
	After correction	2.2419	2.2611	2.3022	2.1944
MRD	Before correction	1.2810	1.2101	1.1657	1.1679
	After correction	1.5678	1.5711	1.5651	1.5513

## Data Availability

All data and code will be made available on request to the correspondent author’s email with appropriate justification.
